# Compound identification of Shuangxinfang and its potential mechanisms in the treatment of myocardial infarction with depression: insights from LC-MS/MS and bioinformatic prediction

**DOI:** 10.3389/fphar.2025.1499418

**Published:** 2025-01-28

**Authors:** Yize Sun, Haibin Zhao, Zheyi Wang

**Affiliations:** ^1^ Department of Traditional Chinese Medicine, Qilu Hospital (Qingdao), Shandong University, Qingdao, Shandong, China; ^2^ Department of Cardiology, Third Affiliate Hospital, Beijing University of Chinese Medicine, Beijing, China; ^3^ Department of Cardiology, Oriental Hospital, Beijing University of Chinese Medicine, Beijing, China; ^4^ Department of Neurology, Qilu Hospital, Cheeloo College of Medicine, Shandong University, Jinan, Shandong, China

**Keywords:** Shuangxinfang, traditional Chinese medicine, myocardial infarction, depresssion, liquid chromatography-mass spectrometry, network pharmacology, molecular docking, neocryptotanshinone

## Abstract

**Background:**

Patients with myocardial infarction (MI) have a high incidence of depression, which deteriorates the cardiac function and increases the risk of cardiovascular events. Shuangxinfang (Psycho-cardiology Formula, PCF) was proved to possess antidepressant and cardioprotective effects post MI. However, the compounds of PCF remain unidentified, and the pertinent mechanism is still not systematic. The purpose of this study is to determine the ingredients of PCF, further to probe the underlying mechanism for MI with depression.

**Methods:**

The compounds of PCF were qualitatively identified by LC-MS/MS. The optimal dosage for lavage with the PCF solution in rats was determined to be 1 mL/100 g/day for a duration of 5 days. We also detected the PCF components migrating to blood in the control and model rats. Then the targets of PCF compounds were searched on Swiss target database, and the targets of depression and MI were predicted on TTD, OMIM, GeneCards, DrugBank and PharmGkb database. All the targets were intersected to construct the Protein-Protein Interaction (PPI) network on Metascape platform and the herb-compound-target (HCT) network on Cytoscape, to identify the hub targets. GO and KEGG pathway enrichment analysis were conducted on DAVID platform. Molecular docking was modeled on AutoDock Vina software.

**Results:**

There were 142 bioactive compounds from PCF acting on 270 targets in a synergistic way. And a total of seven components migrating to blood were identified, including Miltionone I, Neocryptotanshinone, Danshenxinkun A, Ferulic acid, Valerophenone, Vanillic acid and Senkyunolide D. Then SRC and MAPK3 were obtained as the hub proteins by degree value in PPI network, and P2RY12 was picked out as seed proteins ranked by scores from MCODES. Further analysis of biological process and signaling pathways also revealed the significance of ERK/MAPK. Statistical analyses (e.g., GO and KEGG pathway enrichment, PPI network analysis) demonstrated the significance of the identified targets and pathways (*p* < 0.05). Molecular docking results showed that the binding energies were all less than −5 kcal/mol. The stability of Neocryptotanshinone possessed the lowest binding energy to MAPK3.

**Conclusion:**

We identified PCF’s bioactive compounds and predicted its therapeutic mechanism for MI with depression using LC-MS/MS and bioinformatics. Key targets SRC, MAPK3, and seed protein P2RY12 were crucial for PCF’s cardio-neuroprotective effects. Neocryptotanshinone showed the strongest binding to MAPK3, suggesting it as a pivotal active ingredient. These findings offer new insights and targets for future research on PCF.

## 1 Introduction

Accumulated evidence has consistently showed that depression can be induced by the aftermath of myocardial infarction (MI), and in turn deteriorates the cardiac prognosis (Wilkowska et al., 2019). The reported prevalence of depression was present in 22.39%–35.46% of patients with MI, which were associated with arrhythmia, decreased heart rate variability, as well as higher morbidity and mortality (Meijer et al., 2011; Feng et al., 2019). Although selective serotonin reuptake inhibitors (SSRIs) generally exert safe effects on patients with cardiovascular disease, which have been proved by clinical studies recently, the prognosis for long-term cardiac outcomes remains controversial (Berkman et al., 2003; Kim et al., 2018). Furthermore, some patients do not respond to SSRIs, and even fewer achieve remission (Behlke et al., 2020). A cross-sectional study which enrolled 1500 MI participants, declared that only 20% depressed patients were currently in therapy (Moise et al., 2020), perhaps due to low motivation, access/cost, the worry about side effects of drugs and other factors. Thus, there is a need for safe and effective alternative to cover the shortage of current therapy.

According to the theory of Traditional Chinese Medicine (TCM), blood stasis not only brings about obstructed meridian vessel, but also produces the stagnation of vital energy, thus characterized by chest pain and mental fatigue, respectively named as “Zhenxintong” (MI) and “Yubing” (depression). Shuangxinfang (Psycho-cardiology Formula, PCF) consists of four kinds of herbs, among which *Salvia miltiorrhiza* Bunge [Lamiaceae; *Salviae miltiorrhizae* radix et rhizoma] and the roots and rhizomes of *Chuanxiong Rhizoma* [Umbelliferae; *Ligusticum chuanxiong* hort] could promote blood circulation to remove blood stasis, while the bulb of *Lilium pumilum* DC [Liliaceae; Lilii Bulbus] and the dried seeds of *Ziziphi Spinosae Semen* [Rhamnaceae; *Ziziphus jujuba* Mill.var.spinosa (Bunge) Hu ex H.F.Chou] could make the subjects vigorous and uplifting. The therapeutic effects of PCF were supported by clinical evidence in coronary heart disease with depression. To be specific, the effective rate of angina pectoris in PCF group was 90.9%, while the rate of the control group was 73.91%. After treatment with PCF, the score of self-rating depression scale (SDS) decreased from 57.23 ± 5.984 to 23.05 ± 4.815 (Hou et al., 2021). Our previous research demonstrated that PCF reduced the inflammatory reaction derived from myocardial ischemia, and changed the expression of neurotransmitters directly by modifying the expression of the 67 KDa isoform of glutamic acid decarboxylase (GAD67) to relieve depression (Wang et al., 2019). Recent studies have also suggested that the anti-inflammatory mechanism of PCF might attribute to the modulating effect of S100A9 protein on microglial activation (Sun et al., 2021). It’s obvious that PCF begins to come into focus as a promising alternative method for psycho-cardiology diseases. However, at present, the pharmacodynamic components of PCF have not been clearly identified, and the mechanism of PCF for MI merging with depression is still non-holistic and unsystematic.

Actually, the exploration of components in herbs has become an emerging trend in the modernization of TCM. For this purpose, UPLC-Q-Exactive Orbitrap MS/MS (LC-MS/MS), which has the advantages of sensitivity and accuracy in biochemical component analysis, is widely used to separate and identify the compounds from the herbs. On the basis of LC-MS/MS data, we integrated network pharmacology and molecular docking to systematically predict targets of PCF for MI with depression, and analyze the potential mechanisms from the perspective of biological network.

## 2 Materials and methods

### 2.1 Reagents and materials

Formic acid, acetonitrile, H_2_O (grade: for HPLC), sample vial and 0.22 μm microporous membrane were all purchased from Thermo Fisher Scientific, Inc (Waltham, United States). All of the remaining reagents were of analytical grade. PCF granules were provided by Beijing Pharmaceutical Co. Ltd., one dosage of which consists of *S. miltiorrhiza Bunge* 20 g, the roots and rhizomes of *Chuanxiong Rhizoma* 12 g, the dried seeds of *Ziziphi Spinosae Semen* 30 g and the bulb of *L. pumilum DC* 30 g. The optimal lavage dose of the PCF solution for rats was 1 mL/100 g/d for 5 days, and the converted intragastric lavage dose has been verified on the efficacy for depression post-AMI in previous research (Wang et al., 2019).

### 2.2 The myocardial infarction rat model and behavioral tests

Male Sprague-Dawley (SD) rats (220 ± 20 g) were acquired from Beijing Vital River Laboratory Animal Technology Co., Ltd., [License No. SCXK (Beijing) 2016–0006]. The rats were randomly assigned to three groups: control group (n = 6), PCF group (n = 6), model group (n = 6). The rats in the model group underwent ligation of the left anterior descending coronary artery. Rats were intraperitoneally injected with penicillin to prevent infection. Rats in the latter two groups were administered intragastrically PCF solution (1 mL/100 g/d) every day for 5 days, while the same volume of distilled water was orally gavaged in the control group postoperatively. As previously reported, we performed open field test (OFT) and forced swimming test (FST) in model group, and the results have been published in the paper (Sun et al., 2021). The experimental procedures and methods complied with the Institutional Animal Care and Use Committee of the University of Chinese Medicine, Beijing, China (Ethical number: BUCM-4-2020091108-3141).

### 2.3 Sample preparation

The stewing granules were crushed in the grinder, then dissolved in 20 mL methanol, vortexed for 30 s, and sonicated in a KQ-250 DE ultrasonic water bath (Kunshan Ultrasonic Instrument Co. Ltd., Jiangsu, China) operating at 40 kHz with an output power of 300 W for 30 min at room temperature. After standing and cooling, PCF solution was filtered through a 0.22 μm microporous membrane and transferred for further analysis. The serum sample (250 μL) was thawed and dissolved in 750 μL methanol, pre-cooled and incubated at 4°C, swirled for 3 min, centrifuged at 14,000 *g* at 4°C for 15 min. Then, the supernatant was removed to be blown by nitrogen to dry. 200 μL methanol solution was added to redissolve, and the sample was swirled, 14,000 g centrifuged at 4°C for 15 min, and poured into a liquid phase vial for further analysis.

### 2.4 LC-MS/MS analysis

Samples were analysed on a Vanquish Flex System (Thermo Scientific) equipped with a AQUITY UPLC C18 column (1.7 μm × 2.1 mm × 100 mm). The sample injection volume was set at 3μL, while the column temperature was set at 55°C and the flow rate was 0.3 mL/min. The mobile phase consisted of deionized water with 0.1% formic acid (A) and acetonitrile (B). The multi-step linear elution gradient procedure was as follows: 0–3 min (0% B), 3–45 min (0%–75% B), 45–45.1 min (75%–0% B), 45.1–50 min (0%B). Mass Spectrometer (MS) detection was performed on a Q Exactive Plus mass spectrometer (Thermo Fisher Scientific, United States) equipped with an electrospray ionization source (ESI). The mass spectrometry conditions were as follows: the capillary temperature, 320°C; ion spray voltage, 3.0 kV (positive) or 3.5 kV (negative). The sheath gas and auxiliary gas were both high-purity nitrogen (purity >99.99%), the flow rates of which were 35 arbitrary units and 10 arbitrary units respectively. The data were collected and processed using the QualBrowser software within the Xcalibur 4.1 suite (Thermo Fisher Scientific). The information on compounds and their molecular formulas was retrieved from various databases, including the Traditional Chinese Medicine System Pharmacology Database (TCMSP, http://lsp.nwu.edu.cn/tcmsp.php), the Traditional Chinese Medicine Integrated Database (TCMID, http://www.megabionet.org/tcmid/), the Bioinformatics Analysis Tool for Molecular Mechanisms of Traditional Chinese Medicine (Batman-TCM databases, http://bionet.ncpsb.org/batman-tcm/), and additional references. This information was then imported into a local database. Material identification of the peaks, containing raw data, was achieved through the utilization of the local database, which was integrated with the Compound Discoverer 3.0 software (Thermo Fisher Scientific). The compounds were identified based on their relative molecular weight, isotope peaks, retention time, and m/z values. The parameter error was carefully set at 10 ppm to ensure accuracy.

### 2.5 Network pharmacology analysis

The candidate targets were searched by the keywords of “myocardial infarction” and “depression” on the therapeutic target database (TTD, http://db.idrblab.net/ttd/), Online Mendelian Inheritance in Man (OMIM, http://www.omim.org/), GeneCards (http://www.genecareds.org/), DrugBank (https://www.drugbank.ca/) and PharmGkb (https://www.pharmgkb.org/). The information of gene symbol was collected, and data only for “*Homo sapiens*” were included. The targets of PCF compounds were predicted on Swiss Target Prediction database (http://www.swisstargetprediction.ch/). The targets of the PCF components were matched with those of MI with depression by Venn diagram in venn package of R software. Then the intersected targets were imported into Metascape platform with the MCODE algorithm (https://metascape.org/gp/index.html) to construct Protein-Protein Interaction (PPI) networks, that were presented after modification by Cytoscape software (Version 3.8.2). The herb-compound-target (HCT) network was also visualized by Cytoscape to interpret the pharmacological mechanisms of PCF. In the HCT network, we selected the core target according to the degree value. The degree represents the number of lines connected to each node, which is used to evaluate the importance of each node in the network. The larger the node, the larger the degree value. Each edge represents the interaction relationship between proteins, and more lines mean closer correlation. We calculated the degree value of a node using the NetworkAnalyzer module in Cytoscape software. To provide functional associations for identified PCF components, gene ontology (GO) terms and Kyoto Encyclopedia of Genes and Genomes (KEGG) pathway enrichments were conducted on Database for Annotation, Visualization and Integrated Discovery (DAVID) Bioinformatics Resources 6.8 online tool (http://david.abcc.ncifcrf.gov/home.jsp/), with a threshold value of *p* < 0.05. Biological processes (BP), molecular functions (MF) and cellular components (CC) were all included to reveal the function of gene targets. Following this, the top 15 GO terms based on gene rich factor and the top 19 pathways were selected for visual analysis. Bar plots and bubble plots were generated using the ggplot2 package in R software.

### 2.6 Molecular docking

A molecular docking study was undertaken to elucidate the binding affinity of the core compounds (ligands) to the hub proteins. The mol2 structures were retrieved from the TCMSP database and subsequently imported into AutoDock Tools 1.5.6 for charge assignment. Following this, rotatable bonds were rotated, and the structures were converted to the pdbqt format. The protein PDB structures were downloaded from the Protein Data Bank (PDB, https://www.rcsb.org/) and dehydrated, and subsequently, hybrid molecules were excised from the protein using PyMOL software. The binding site was utilized to allocate grid boxes for the targets, ensuring ample space for the translational and rotational movements of the ligands. The receptors underwent hydrogenation and charge addition in AutoDock Tools, ultimately being saved in the pdbqt format as well. The molecular docking of the protein and ligand was carried out in AutoDock Vina 1.1.2, and visualizations were facilitated by the utilization of PyMOL software and Discovery Studio 2020.

## 3 Results

### 3.1 Identification of components in PCF

**FIGURE 1:**
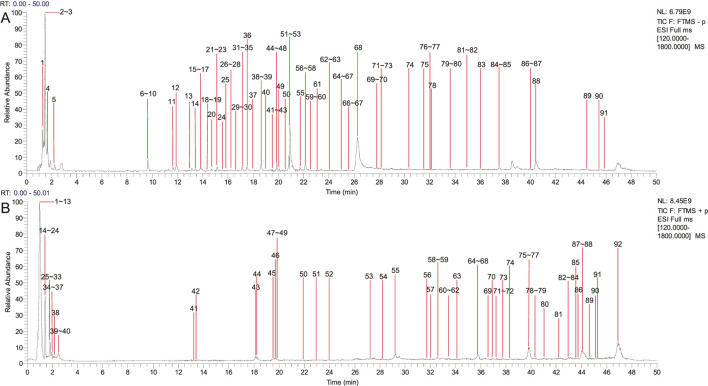
Compounds characterization of PCF by LC-MS/MS. **(A)** Typical base peak chromatographs in the negative ion mode. **(B)** Typical base peak chromatographs in the positive ion mode.

The chemical components from PCF were well separated and detected in both positive and negative ion models. Separately, a total of 91 chemical constituents were identified under negative ion mode (Supplementary Table S1; Figure 1A), while 92 compounds were identified in positive ion mode (Supplementary Table S2; Figure 1B). The typical base peak chromatographs were shown in Figure 1. After removing the repetition, 142 chemical components were analyzed, 74 of which from *S. miltiorrhiza* Bunge, 40 from *Chuanxiong Rhizoma*, 14 from the dried seeds of *Ziziphi Spinosae Semen* and 18 from the bulb of *L. pumilum* DC.

### 3.2 Identification of components of PCF in blood

**FIGURE 2:**
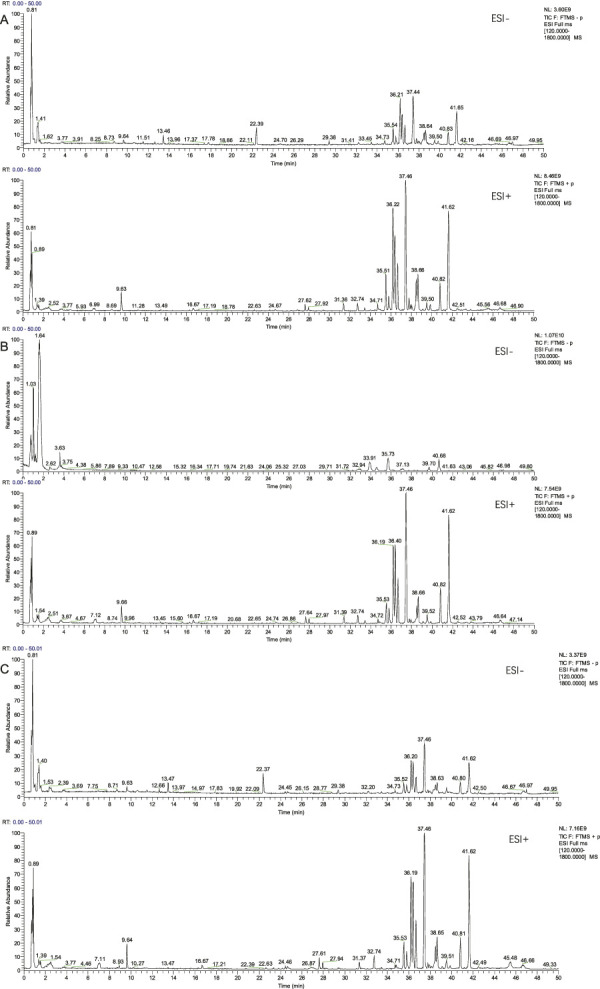
Typical basal peak ion flow patterns of serum samples from different groups. **(A)** Typical basal peak ion flow patterns of serum samples in control group. **(B)** Typical basal peak ion flow patterns of serum samples in PCF group. **(C)** Typical basal peak ion flow patterns of serum samples in model group.

A total of seven components were identified as components in serum, among which, two compounds were found and identified as Miltionone I and Neocryptotanshinone in PCF group. These seven compounds, including Miltionone I, Neocryptotanshinone, Danshenxinkun A, Ferulic acid, Valerophenone, Vanillic acid and Senkyunolide D, were constituents migrating to blood in model. The molecular formula and structure of compounds were listed in Table 1. The typical basal peak ion flow patterns of serum samples from different groups were shown in Figure 2.

**TABLE 1:** A total of seven components of PCF were identified in blood.

No.	PubChem CID	Compound	Molecular formula	Structure	PCF	Model
1	5319835	Miltionone I	C_19_H_20_O_4_	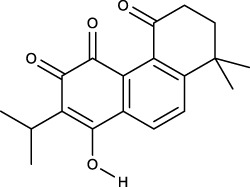	ESI+	ESI+
2	389888	Neocryptotanshinone	C_19_H_22_O_4_	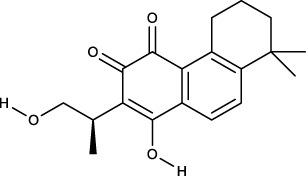	ESI+	ESI+
3	149138	Danshenxinkun A	C_18_H_16_O_4_	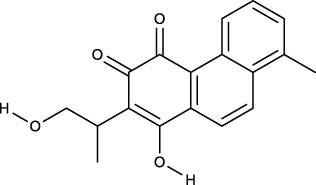	ESI−	ESI+
4	445858	Ferulic Acid	C_10_H_10_O_4_	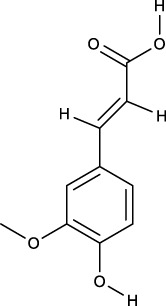	ESI−	ESI+
5	66093	Valerophenone	C_11_H_14_O	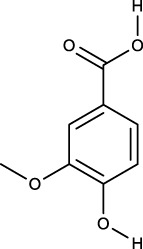	ESI−	ESI+
6	8468	Vanillic acid	C_8_H_8_O_4_	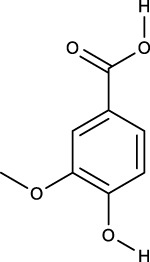	ESI−	ESI+
7	11264524	senkyunolide-D	C_12_H_14_O_4_	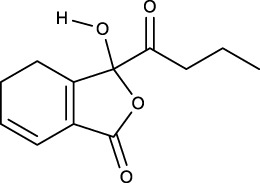	ESI−	ESI+

ESI+: positive ion mode, ESI−: negative ion mode.

### 3.3 Herb-compound-target (HCT) network analysis

**FIGURE 3:**
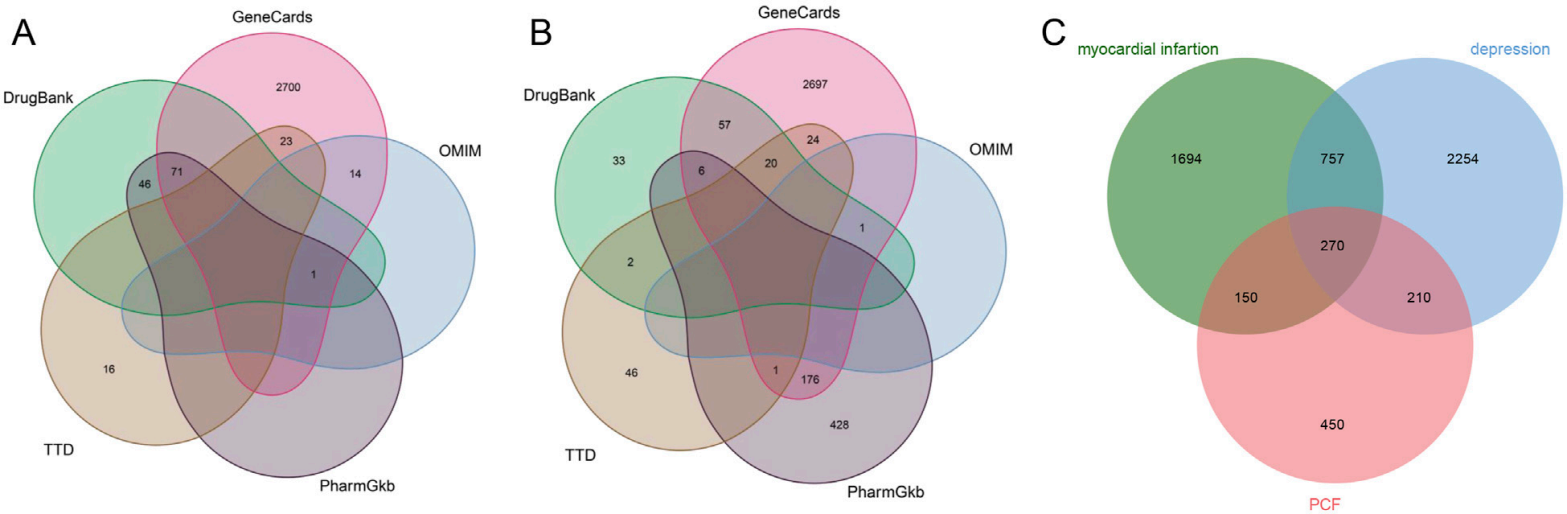
Venn diagram. **(A)** Venn diagram of 2,817 candidate targets for myocardial infarction searched from TTD, OMIM, GeneCards, DrugBank and PharmGkb database. **(B)** Venn diagram of 3491 candidate targets for depression identified from TTD, OMIM, GeneCards, DrugBank and PharmGkb database. **(C)** 270 targets obtained by Venn diagram of intersection for disease targets and PCF targets.

**FIGURE 4:**
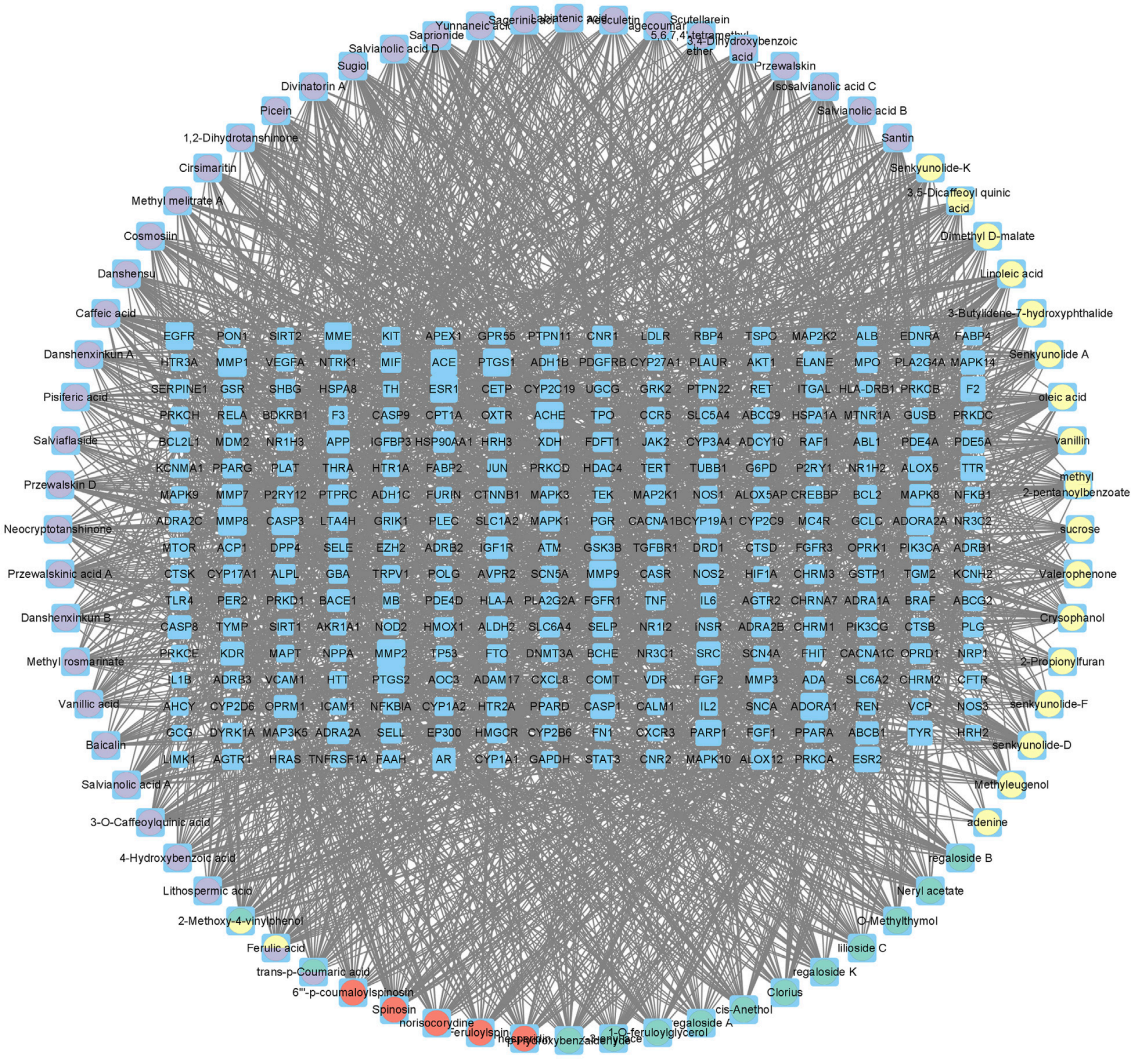
Herb-compound-target (HCT) network. The blue rectangular nodes represent the common targets, and the round nodes represent the components.

There were 2,817 candidate targets of MI (Figure 3A) and 3491 targets of depression identified (Figure 3B). Also, a total of 1,080 targets of PCF compounds were discovered. Then the Venn diagram was conducted to intersect the targets of PCF components with the disease targets, and 270 proteins were generated (Figure 3C). The 270 common proteins were regarded as potential targets of PCF against MI with depression. Moreover, we constructed a connection network of herb-compound-target on Cytoscape platform (Figure 4). There were 342 nodes and 2,435 edges obtained in the HCT network.

### 3.4 Protein-protein interaction network analysis

**FIGURE 5:**
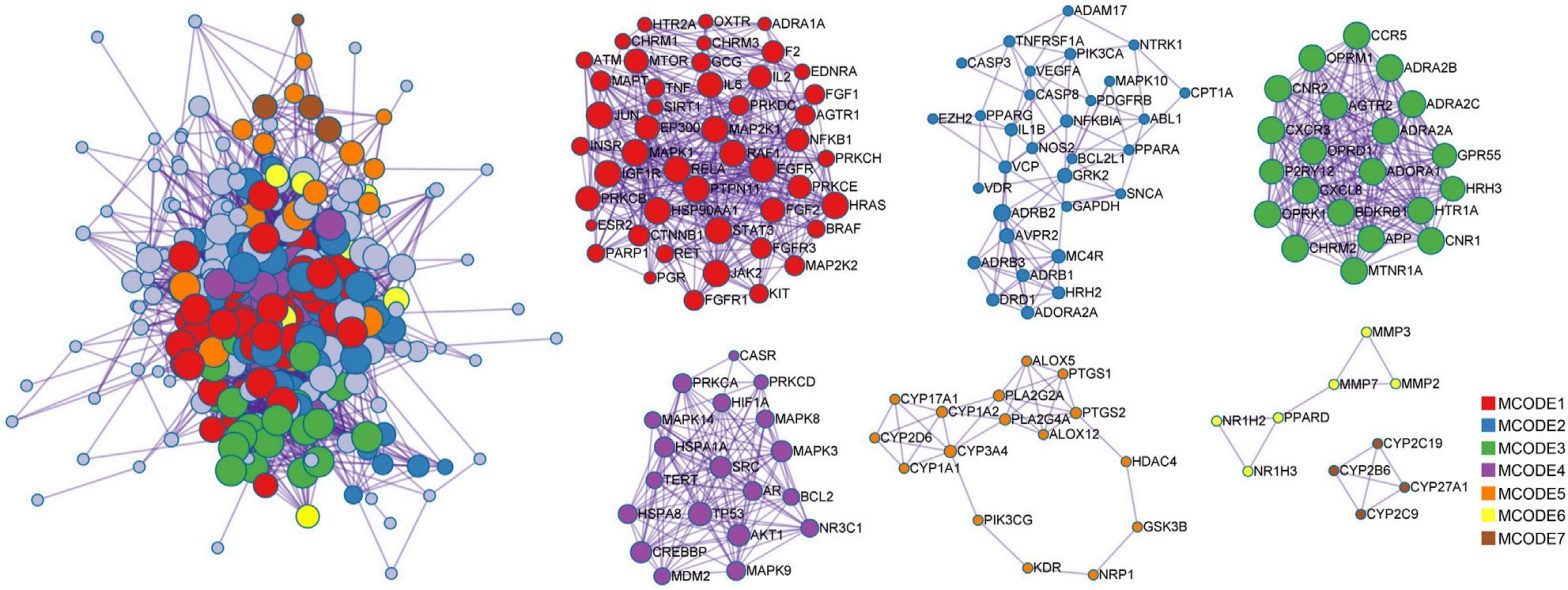
Protein-protein interaction network for 270 overlapping targets. Seven MCODES are presented as functional clustering of protein interaction networks. The lines between targets stand for the predicted relationship, and the degree value is determined by the number of lines. The sizes of the nodes are illustrated from large to small in descending order of degree values.

PPI network analysis was performed on STRING platform for 270 intersection proteins to obtain 5,496 edges in Figure 5. The degree value was reflected by the number of lines connected to each node to evaluate the interaction of proteins in the network. According to the ranking of degree value, the top 15 hub proteins were presented in Table 2, among which, SRC and MAPK3 showed as the targets with the highest degree value. The mean value of nodes in degree was 9.83, and the degree of 130 proteins exceeded the average level. Also, betweenness centrality was listed and measured the extent to which a node lies on paths between other nodes. Subsequently, MCODE plug-in was used to confirm the PPI network function clusters. MCODE 1 (score: 7.96) contained 47 nodes, and the seed protein was PGR. MCODE 2 (score: 2.74) consisted of 31 nodes and the seed protein was NTRK1. MCODE 3 (score: 9.86) included 21 nodes, and the seed protein was P2RY12. MCODE 4 (score 6.21) consisted of 19 nodes, and the seed protein was MAPK8. MCODE 5 (score 2.12) consisted of 16 nodes, and the seed protein was PTGS2. MCODE 6 (score 1.67) and MCODE 7 (score 1.50) consisted of six nodes and four nodes, and the seed proteins were MMP3 and CYP2B6, respectively.

**TABLE 2:** The top 15 targets of PCF for MI and depression in PPI network.

Number	Target gene	Degree	Betweenness centrality
1	SRC	100	0.089595222
2	MAPK3	98	0.044884601
3	TP53	96	0.123922841
4	MAPK1	94	0.041991552
5	STAT3	92	0.063186147
6	HSP90AA1	86	0.060906895
7	EP300	80	0.047569013
8	JUN	80	0.039589893
9	PIK3CA	78	0.045403983
10	RELA	78	0.029370548
11	AKT1	72	0.029710154
12	HRAS	72	0.022310509
13	CREBBP	70	0.080191328
14	ESR1	68	0.052785389
15	CTNNB1	62	0.038781916

### 3.5 Enrichment analysis of mapped targets

**FIGURE 6:**
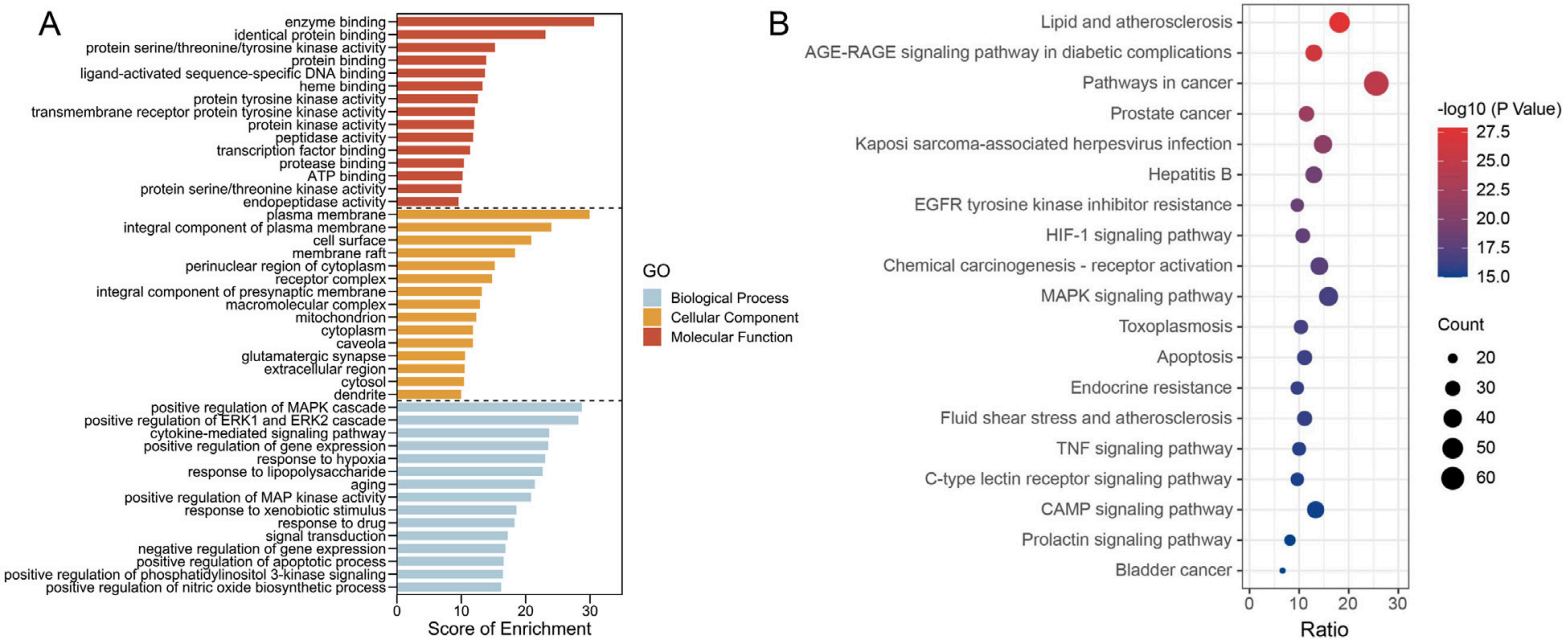
The enrichment results present GO terms and KEGG pathways. **(A)** The top 10 (ranked by p-value) GO terms across three categories (Biological process, Cellular component, Molecular function). **(B)** The top 20 KEGG pathways. The p-values are indicated by the colors of the dots, and the counts are shown by the size of the dots.

To elucidate the biological function of PCF against MI with depression, the enrichment analysis of 270 targets was conducted. After filtering with *p* < 0.05 as threshold, 1128 GO items and 174 pathways were identified, among of which, the top 15 enriched GO items (Figure 6A) and the top 19 KEGG pathways (Figure 6B) were visualized as follows. For the MF analysis, the targets were mainly involved in enzyme binding, identical protein binding, protein serine/threonine/tyrosine kinase activity, protein binding, ligand-activated sequence-specific DNA binding, heme binding, protein tyrosine kinase activity, transmembrane receptor protein tyrosine kinase activity, protein kinase activity, peptidase activity, transcription factor binding, protease binding, ATP binding, protein serine/threonine kinase activity, and endopeptidase activity. Cell components were mainly enriched in plasma membrane, integral component of plasma membrane, cell surface, membrane raft, perinuclear region of cytoplasm, receptor complex, integral component of presynaptic membrane, macromolecular complex, mitochondrion, cytoplasm, caveola, glutamatergic synapse, extracellular region, and cytosol and dendrite. Biological processes mainly included positive regulation of MAPK cascade, positive regulation of ERK1 and ERK2 cascade, cytokine-mediated signaling pathway, positive regulation of gene expression, response to hypoxia, response to lipopolysaccharide, aging, positive regulation of MAP kinase activity, response to xenobiotic stimulus, response to drug, signal transduction, negative regulation of gene expression, positive regulation of apoptotic process, positive regulation of phosphatidylinositol 3-kinase signaling and positive regulation of nitric oxide biosynthetic process.

The pathways were mainly enriched in lipid and atherosclerosis, AGE-RAGE signaling pathway in diabetic complications, pathways in cancer, prostate cancer, kaposi sarcoma-associated herpesvirus infection, hepatitis B, EGFR tyrosine kinase inhibitor resistance, HIF-1 signaling pathway, Chemical carcinogenesis - receptor activation, MAPK signaling pathway, toxoplasmosis, apoptosis, endocrine resistance, fluid shear stress and atherosclerosis, and TNF signaling pathway.

### 3.6 Molecular docking

**FIGURE 7:**
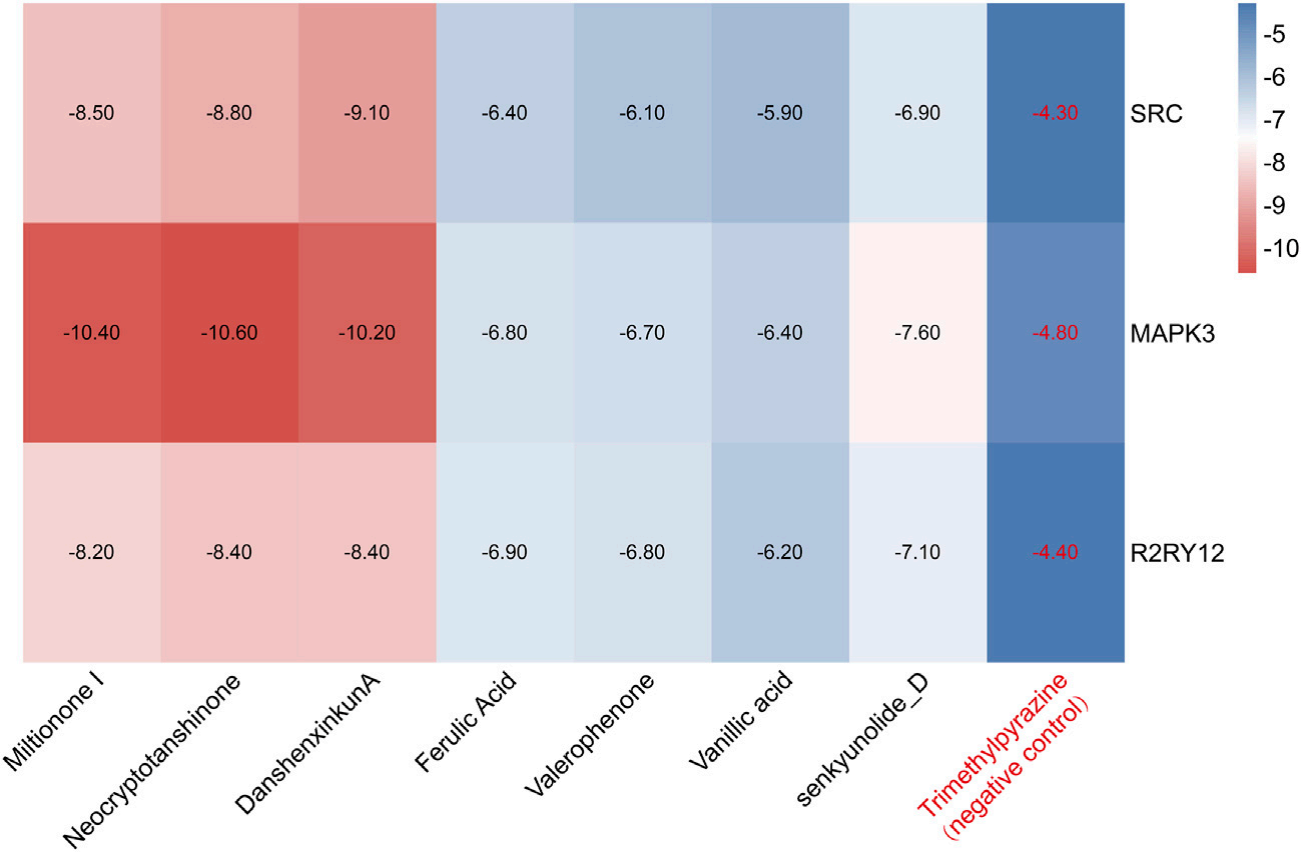
The heat map of molecular docking results. The blue indicates a high docking score, and the red indicates a low docking score. The lower the docking score, the more stable the bond.

**FIGURE 8:**
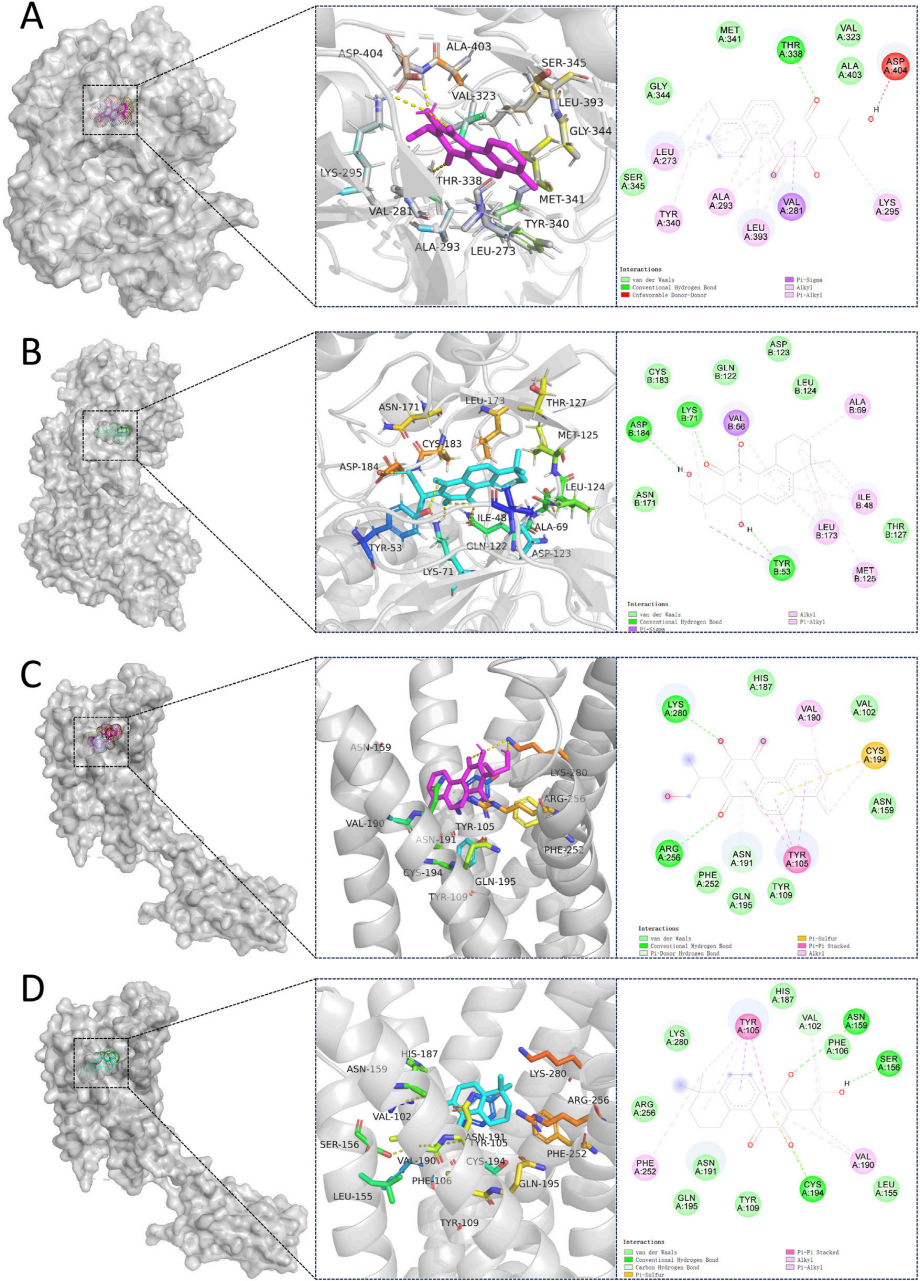
Molecular docking between 7 natural compounds from PCF and 3 targets for myocardial infarction with depression. The 2D and 3D structures of the protein targets are shown in figure. **(A)** Docking mode of SRC-Danshenxinkun A. **(B)** Docking mode of MAPK3-Neocryptotanshinone. **(C)** Docking mode of P2RY12-Danshenxinkun A. **(D)** Docking mode of P2RY12-Neocryptotanshinone.

We performed molecular docking on components in serum, including Miltionone I, Neocryptotanshinone, Danshenxinkun A, Ferulic acid, Valerophenone, Vanillic acid, and Senkyunolide D, along with Trimethylpyrazine, a non-active ingredient from *Chuanxiong Rhizoma*, as a negative control. It’s generally acknowledged that lower binding energy predicts better stability of the ligands to the receptors and stronger bonding for interactions. However, for Trimethylpyrazine, the predicted binding energy as a negative control was greater than −5 kcal/mol. The binding energies for the other components were all negative and less than −5 kcal/mol, which are listed in Figure 7. The free binding energy of Danshenxinkun A with SRC (PDB id-2src) was −9.1 kcal/mol. The binding affinity was contributed by the following: the hydrogen bonding with the THR338 residue; the van der Waals forces with the SER-345, GLY-344, MET-341, VAL-323 and ALA-403 residues; the Pi–Sigma interaction with the VAL-281 residue; the Pi-Alkyl interaction with the LEU-273, TYR-340, ALA-293, LEU-393 and LYS-295 residues (Figure 8A). The free binding energy of Neocryptotanshinone with MAPK3 (PDB id-4qtb) was −10.6 kcal/mol. The binding affinity was contributed by the following: the hydrogen bonding with the ASP-184, LYS-71 and TYR-53 residues; the van der Waals forces with the ASN-171, LYS-183, GLN-122, ASP-123, THR-127 and LEU-124 residues; the Pi-Sigma interaction with VAL-56 residue; the Pi–Alkyl stacking interactions with the ALA-69, ILE-48, LEU-173 and MET-125 residues (Figure 8B). The free binding energy of Danshenxinkun A with P2RY12 (PDB id-4ntj) was −8.4 kcal/mol. The binding affinity was contributed by the following: the hydrogen bonding with the CYS-280 and ARG-256 residues; the van der Waals forces with the HIS-187, ASN-159, VAL-102, TYR-109, ASN-191, GLN-195 and PHE-252 residues; the Pi–Pi stacking interaction with the TYR-105 residue; the Alkyl interaction with the VAL-190 residue (Figure 8C). The free binding energy of Neocryptotanshinone with 2RY12 (PDB id-4ntj) was −8.4 kcal/mol. The binding affinity was contributed by the following: the hydrogen bonding with the CYS-194, ASN-159 and SER-156 residues; the van der Waals forces with the ARG-256, LYS-280, HIS-187, VAL-102, PHE-106, LEU-155, TYR-109, ASN-191 and GLN-195 residues; the carbon hydrogen bonding with the VAL-102 residue; the Pi-Pi stacking interaction with TYR-105 residues; the Pi–Alkyl stacking interactions with the VAL-190 and PHE-252 residues (Figure 8D). The results showed that, in the protein-ligand binding of this study, the proton and oxygen of the hydroxyl and the ketonic oxygen tend to form hydrogen bond with the active site residues of the proteins, while the benzene rings tend to interact with the proteins by aromatic stacking and Van der Waals forces.

## 4 Discussion

In this study, the compounds of PCF were determined by LC-MS/MS, and the therapeutic mechanism for MI with depression was predicted by network pharmacology. Consequently, a total of 142 compounds from PCF and seven compounds in serum were identified. Moreover, 270 targets for MI with depression and drugs were intersected. Then enrichment analysis and PPI network were performed. Also, hub proteins were obtained by degree value and protein clustering.

The seven components migrating to blood included Miltionone I, Neocryptotanshinone, Danshenxinkun A, Ferulic acid, Valerophenone, Vanillic acid and Senkyunolide D. Molecular docking was conducted to further validate the binding activities between the components in blood and hub proteins. The binding energies were all less than −5 kcal/mol, which indicated that the ingredients could combine with SRC, MAPK3 and P2RY12, respectively. Additionally, the stability of Neocryptotanshinone, Danshenxinkun A, and Miltionone I to the selected hub targets was much more reliable than that of the other compounds. Interestingly, among the selected compounds, Neocryptotanshinone possessed the lowest binding energy to most of the targets, especially to MAPK3. It’s implied that Neocryptotanshinone probably reacted as the pivotal active ingredient of PCF in the treatment of MI with depression. As early as 1987, designated Neocryptotanshinone have been isolated from “Tan-Shen”, the root of Salvia miltiorrhiza, by spectral and physical data (Lee et al., 1987). Wu et al. found that Neocryptotanshinone has anti-inflammatory effects in LPS-stimulated mouse macrophages (RAW264.7) by down-regulating the expression of iNOS and NF-κB p65 (Wu et al., 2015). Recently, Neocryptotanshinone was reported to promote the autolysosome removal of protein aggregation to exert the therapeutic advantages of myocardial ischemia reperfusion injury via the ERK1/2-Nrf2-LAMP2 signaling pathway (Yang et al., 2023). Ma et al. found that Neocryptotanshinone improved myocardial energy metabolism in mice with HF post-AMI via regulating the RXRα/PPARα pathway (Ma et al., 2023). In addition, compared with other compounds, the binding of Danshenxinkun A to SRC was slightly more stable. However, we have not searched the studies on the effects of Neocryptotanshinone or Danshenxinkun A with regard to psycho-cardiology diseases up to now. It means that the studies for these two ingredients have not yet started, thus, huge spaces currently exist to further develop.

Relatively much more research have been performed in Ferulic acid and Vanillic acid for MI. Ferulic acid, a derivative of cinnamic acid and a natural phenolic acid compound, has antioxidant and anti-inflammatory properties (Zduńska et al., 2018). Zhang et al. found that Ferulic acid can reduce the colocalization of Kelch-like ECH-associated Protein 1 (Keap1) and Nuclear Factor E2-related Factor 2 (Nrf2), and inhibit the cardiomyocyte apoptosis by alleviating oxidative stress (Zhang et al., 2021). In the mouse model of acute myocardial infarction, Ferulic acid can also inhibit the apoptosis of H9C2 cells induced by TNF-α or actinomycosterone via blocking ROS production and Caspase-3 activity (Li et al., 2020). Vanillin is the oxidized form of vanillin produced during the conversion of vanillin to Ferulic acid (Stanely Mainzen Prince et al., 2011). At present, experiments have provided evidence for the effectiveness of Vanillic acid for cardiovascular diseases. Ponnian et al. evaluated the preventive effect of Vanillic acid (5 mg/kg, 10 mg/kg) on myocardial infarction rats induced by isoproterenol, and found that vanillic acid not only reduced the infarct size, but also downregulated the expression of bcl-2 and bcl-2 associated X (Bax) gene (Prince et al., 2011). In the H9C2 cells hypoxia/reoxygenation model, vanillin pretreatment was found to significantly improve cell viability, reduce the activities of lactate dehydrogenase and creatine phosphokinase in the supernatant, and recover the mitochondrial membrane potential (Yao et al., 2020). The antidepressant mechanism of Ferulic acid has also been extensively explored. Kazunori et al. found that continuous administration of Ferulic acid (5 mg/kg) for 7 days significantly reduced the duration of tail suspension in stressed mice, which may attribute to the increasing levels of dopamine and noadrenaline, brain-derived neurotrophic factor and ATP. The research suggests that Ferulic acid may play an antidepressant role by enhancing nerve cell survival and proliferation, energy metabolism and dopamine synthesis (Sasaki et al., 2019). Moreover, abdominal injection of Ferulic acid (12.5, 25 and 50 mg/kg/day) for 28 consecutive days in stressed rats significantly increased sucrose intake, reduced immobility time in forced swimming test, and increased the expression of glucocorticoid receptor (Zheng et al., 2019). In CUMS mice models, Ferulic acid can improve depression-like behavior by inhibiting microglial overactivation, NF-κB signaling, and NLRP3 inflammatome activation (Liu et al., 2017). Our previous *in vitro* studies also confirmed the anti-inflammatory effects of Ferulic acid (Sun et al., 2022).

To annotate the biological functions of 270 targets, the GO enrichment analysis was performed. The 15 enriched BP terms were mainly concentrated on MAPK cascade, ERK 1/2 cascade, cytokines, response to hypoxia, apoptosis, and nitric oxide biosynthesis. The KEGG results implied that the pathways with relatively high counts were intimately connected with atherosclerosis, tumor, virus infection, HIF-1 signaling pathway, MAPK signaling pathway, TNF signaling pathway, and cAMP signaling pathway. Remarkably, the degree value of MAPK3 also showed among the top few in PPI network. On the one hand, it’s implied that PCF formulas with four kinds of herbs can react through the multi-targets and multi-pathway mechanism. On the other hand, integrated with the above results, it’s obvious that ERK/MAPK signal was vital importance, thus deserved further analyzation hereinafter. MAPK 3, also named extracellular-signal regulated kinase (ERK) 1, as member from the subfamilies of mitogen-activated protein kinases (MAPKs), is responsible for cell proliferation, apoptosis, neurogenesis, as well as neural differentiation, migration, the final fate, and plastic changes. The ERK pathway receives signals from two main receptors, including G-protein coupled receptor (GPCR) and tyrosine kinase (RTK). The two receptor-related signaling pathways were also enriched in this study. Brain-derived neurotrophic factor (BDNF) and cAMP response element-binding protein (CREB), exist as the upstream and downstream regulator of ERK respectively, also express in parallel to that of ERK (Wang and Mao, 2019). ERK manages in fear conditioning, social and emotional behavior, and subserves memory processes, such as long-term potentiation (Albert-Gascó et al., 2020). There is an abundance of evidence to support the model that the ERK pathway, acting as a substrate of antidepressants, is downregulated in the prefrontal cortex and hippocampus (Qi et al., 2006; Meller et al., 2003). ERK 1/2 is entitled an intriguing name, the reperfusion injury salvage kinase (RISK), which confers cardioprotection during myocardial reperfusion (Hausenloy and Yellon, 2007).By the way, the mitochondrial permeability transition pore (mPTP) opens in the first few minutes after myocardial ischemia/reperfusion injury, and ERK1/2 pathway, considered to regulate mitochondrial permeability transition thereby inhibiting cysteinyl aspartate specific proteinase (Caspase) activation, is protective for cardiac function (Xie et al., 2014). The ERK/MAPK pathway, with the upstream receptors and downstream regulatory elements, appeared in the enrichment results, indicating that ERK pathway provides a significant way for PCF to exert therapeutic effect on MI with depression.

In our study, SRC was obtained as the central protein by degree value in PPI network. SRC, a non-receptor tyrosine kinase of SRC family kinase (SFK), is abundant at synaptic sites to demonstrate an important role in depressive disorder (Wang et al., 2022). Depression can be influenced by SFK for the function of phosphorylation for N-methyl-d-aspartate (NMDA) receptors. The level of SRC was reduced in mice model of *postpartum* depression, that subsequently decreased tyrosine phosphorylation of NMDA receptors and impaired the hippocampal neurogenesis (Zhang et al., 2016). Therefore, the administration of the SRC inhibitor led to depression-like behavior in hormone-stimulated pregnancy mice (Zhang et al., 2016). The paradox was that forced swimming could cause tyrosine phosphorylation of signal regulatory proteinα (SIRPα) in the mice brain by the activation of SRC family kinases and depression-like behavior in reply to stress (Ohnishi et al., 2010). The role of SRC in cardiovascular diseases was also observed by some researches. SRC was proved to be activated in MI models, on the other hand, the inhibitors of SRC reduced the occurrence arrhythmias and sudden cardiac death in mice with a cardiac-specific activated renin-angiotensin system (Zheng et al., 2020).

P2RY12 was picked out as seed proteins ranked by scores from MCODES. P2RY12, as one of the members of P2Y receptor group (subfamilies of purinergic receptors), can be activated by purines and pyrimidines. It’s well established that P2RY12 is expressed on platelets and the ramified processes of microglia, which plays a fundamental role in platelet activation and microglial function. In response to the activation of adenosine diphosphate (ADP), P2RY12 triggers platelet aggregation and thromboembosis (Herbert, 2004). Thereby, the P2RY12 receptor inhibitors in pharmacotherapy have been recommended for the prevention and treatment of cardio-cerebrovascular diseases (Jacobson et al., 2020). According to the literature, P2RY12 receptor is necessary to maintain lipophagic flux and cholesterol homeostasis of vascular smooth muscle cells, and the regulation in advanced atherosclerosis attributes to the inhibition of autophagy through the PI3K-AKT-mTOR signaling pathway (Pi et al., 2021). Microglia acting as immune sentinels and expressing purine receptors on cell-surface, the involvement of which in migration and phagocytosis is controlled by P2RY12 signaling. At the time of microglia being activated, the expression of P2RY12 is decreased, indicating a transform in the functional pattern from chemotactic to phagocytic (Koizumi et al., 2013). The recent research showed that extracellular ADP activated NF-κB and the NOD-like receptor protein 3 (NLRP3) inflammasome to enhance microglial inflammation via the P2Y12 receptor (Suzuki et al., 2020). Microglia, which triggered neuroinflammation via the release of proinflammatory cytokines in depression, has attracted a lot of attraction. Wu et al. implied that the decrease in P2Y12 receptors might aggravate the damage of hippocampal neurogenesis in the chronic unexpected mild stress (CUMS) mice with severe depression-like behavior (Wu et al., 2021). Interestingly, ERK1/2 and SRC have also been substantiated to modulate microglial chemotaxis. Activation of ERK1/2 and SRC in response to the stimulation of P2RY12 separately results in the phosphorylation of Paxillin at Ser83 and Tyr31, which is required for adhesion disassembly during chemotaxis (Gómez Morillas et al., 2021). Coincidentally, atherosclerosis and platelet activation severally occurred as results of pathway enrichment and biological process in this study. Besides, our previous study declared that PCF suppressed microglia inflammation to alleviate depression-like behavior. It’s implicated that P2RY12-mediated cardiovascular thrombosis and cerebral microglia activation may provide potential targets for MI with depression, that deserves much more attention in the research of psycho-cardiology diseases.

Our study is indeed subject to several noteworthy limitations. Firstly, our focus and screening for virtual docking were primarily narrowed down to targets with high degree values in the context of network pharmacology analysis, potentially overlooking a considerable share of other potential targets. Secondly, although our study was primarily rooted in data analysis, further experimental validation remains pivotal in enhancing the credibility of our findings. This validation may encompass employing techniques such as isothermal titration calorimetry and microscale thermophoresis to delve into the interactions between compounds and proteins, as well as the regulatory effects of blood-borne constituents on MI coupled with depression via the ERK/MAPK pathway. Additionally, the scarcity of diverse blood collection time points in this study renders pharmacokinetic data, including bioavailability, unobtainable. Although we have evaluated the efficacy of PCF at a specific dose, the ED50 has not been determined in this study. Therefore, we emphasize the need for further research to investigate the dose-response relationship of PCF and its individual components.

## 5 Conclusion

Taken together, we applied LC-MS/MS technology, network pharmacology, and molecular docking to comprehensively identify the components of Shuangxinfang (PCF) and decipher the potential therapeutic targets for myocardial infarction (MI) with depression. A total of 142 bioactive compounds from PCF were identified, acting on 270 targets in a synergistic manner. Seven components were found to migrate to the blood, including Miltionone I, Neocryptotanshinone, Danshenxinkun A, Ferulic acid, Valerophenone, Vanillic acid, and Senkyunolide D. Network pharmacology analysis revealed SRC and MAPK3 as hub targets, while P2RY12 was identified as a seed protein. Further analysis showed the significance of the ERK/MAPK signaling pathway in the cardio-neuroprotective effects of PCF. Molecular docking results indicated that Neocryptotanshinone possessed the lowest binding energy to MAPK3, suggesting its pivotal role as an active ingredient. These findings provide pharmacological ingredients and target references for further scientific research and potential clinical applications in treating MI with depression. However, our study is subject to limitations, such as the focus on high-degree targets in virtual docking and the lack of experimental validation. Future research should aim to address these limitations and further elucidate the therapeutic mechanism of PCF.

## Data Availability

The original contributions presented in the study are publicly available. This data can be found here: https://doi.org/10.6084/m9.figshare.27109966.v1; https://doi.org/10.6084/m9.figshare.27109969.v1; https://doi.org/10.6084/m9.figshare.27110107.v1; https://doi.org/10.6084/m9.figshare.27110110.v1; https://doi.org/10.6084/m9.figshare.27110113.v1; https://doi.org/10.6084/m9.figshare.27110116.v1; https://doi.org/10.6084/m9.figshare.27110119.v1; https://doi.org/10.6084/m9.figshare.27110122.v1.

## Glossary

ADP: adenosine diphosphate
BDNF: brain-derived neurotrophic factor
BP: biological processes
Caspase: cysteinyl aspartate specific proteinase
CC: cellular components
CREB: cAMP response element-binding protein
CUMS: chronic unexpected mild stress
DAVID: database for annotation, visualization and integrated discovery
DG: dentate gyrus
ERK: extracellular-signal regulated kinase
GAD67: the 67 KDa isoform of glutamic acid decarboxylase
GO: gene ontology
GPCR: G-protein coupled receptor
HCT: herb-compound-target
HO-1: heme Oxygenase-1
KEGG: kyoto encyclopedia of genes and genomes
LC-MS/MS: UPLC-Q-Exactive Orbitrap MS/MS
MAPK: mitogen-activated protein kinase
MF: molecular functions
MI: myocardial infarction
mPTP: mitochondrial permeability transition pore
NMDA: N-methyl-d-aspartate
NF-κB: nuclear factor kappa-B
NLRP3: NOD-like receptor protein 3
Nrf2: nuclear factor E2-related factor 2
PGC1α: peroxisome proliferator-activated receptor-γ co-activator-1α
PUFAs: polyunsaturated fatty acids
RISK: reperfusion injury salvage kinase
RTK: tyrosine kinase
SFK: SRC family kinase
SIRPα: signal regulatory proteinα
SSRI: sselective serotonin reuptake inhibitors
TCM: Traditional Chinese Medicine
