# Supplementary material for: Compound identification of Shuangxinfang and its potential mechanisms in the treatment of myocardial infarction with depression: insights from LC-MS/MS and bioinformatic prediction
Source: Front Pharmacol. 2025 Jan 28;16:1499418. doi: 10.3389/fphar.2025.1499418 (PMC11811099; doi:10.3389/fphar.2025.1499418)
Supplement: Supplementary file 1 [file Table1.docx]

Table S1. PCF compounds in the negative ion mode.

| Peaks  no. | tR/min | Molecular formula | Detection mode | Theoretical value | Measured value | Secondary  fragment（MS/MS） | error（ppm） | ingredients | resource |
| --- | --- | --- | --- | --- | --- | --- | --- | --- | --- |
| 1 | 1.41 | C_6_H_10_O_5_ | [M-H] | 161.0455 | 161.0459 | 142.05116，131.03517，85.02968，73.02960 | 9.067 | Dimethyl D-malate | CX |
| 2 | 1.48 | C_9_H_18_O_8_ | [M-H] | 253.0929 | 253.0926 | 133.01451，115.00391，96.96026 | 3.224 | lilioside C | BH |
| 3 | 1.48 | C_12_H_22_O_11_ | [M-H] | 341.1089 | 341.1096 | 179.05643，119.03518，89.02463，59.01398 | 5.137 | sucrose | CX |
| 4 | 1.58 | C_21_H_18_O_11_ | [M-H] | 445.0776 | 445.0858 | 220.97643，102.95701 | 1.99 | Baicalin | DS |
| 5 | 2.2 | C_5_H_5_N_5_ | [M-H] | 134.0472 | 134.0474 | 107.03648，92.02564 | 9.834 | adenine | CX |
| 6 | 9.63 | C_8_H_8_O_2_ | [M-H] | 135.0452 | 134.8656 | 92.02534，69.20502 | 0.77 | Clorius | BH |
| 7 | 9.63 | C_20_H_18_O_10_ | [M-H] | 418.0827 | 417.0802 | 219.02797，197.04593，179.03526 | -3.484 | Salvianolic acid D | DS |
| 8 | 9.63 | C_9_H_10_O_5_ | [M-H] | 197.0455 | 197.0459 | 179.03523，135.04538，123.04535，72.99321 | 7.359 | Danshensu | DS |
| 9 | 9.65 | C_8_H_8_O_3_ | [M-H] | 151.0401 | 151.0403 | 136.01688，123.04538，108.02180 | 8.868 | vanillin | CX |
| 10 | 9.82 | C_7_H_6_O_4_ | [M-H] | 153.0193 | 153.0196 | 109.02967，78.98603 | 9.05 | 3,4-Dihydroxybenzoic acid | DS |
| 11 | 11.73 | C_16_H_19_NO_8_ | [M-H] | 352.1038 | 352.1046 | 232.11957，188.07205，146.06142，96.96025 | 5.53 | N-glc-indoleacectic Acid | SZR |
| 12 | 11.9 | C_7_H_6_O_3_ | [M-H] | 137.0244 | 137.0246 | 109.02991，93.03444 | 9.629 | 4-Hydroxybenzoic acid | DS |
| 13 | 12.94 | C_15_H_14_O_6_ | [M-H] | 289.0718 | 289.0725 | 245.08235，203.07184，179.03528，125.02459 | 6.28 | (-)-Epicatechin | BH |
| 14 | 13.42 | C_16_H_18_O_9_ | [M-H] | 353.0878 | 353.0958 | 191.05655，179.03532，173.04582，135.04544 | 1.88 | 3-O-Caffeoylquinic acid | DS |
| 15 | 13.8 | C_7_H_8_O_2_ | [M-H] | 123.0452 | 123.0454 | 121.02950，95.05042，69.89320 | 10.679 | 2-Propionylfuran | CX |
| 16 | 13.8 | C_8_H_8_O_4_ | [M-H] | 167.035 | 167.0353 | 123.04540，95.05044 | 8.171 | Vanillic acid | DS |
| 17 | 13.92 | C_9_H_8_O_4_ | [M-H] | 179.035 | 179.0353 | 135.04541，107.05068 | 7.791 | Caffeic acid | DS |
| 18 | 14.37 | C_18_H_24_O_11_ | [M-H] | 415.1246 | 415.1255 | 179.03526，161.02467，135.04539 | 4.751 | regaloside K | BH |
| 19 | 14.38 | C_7_H_6_O_2_ | [M-H] | 121.0295 | 121.0297 | 120.05660，108.02198，93.03473 | 10.692 | p-Hydroxybenzaldehyde | BH |
| 20 | 14.69 | C_19_H_21_NO_4_ | [M-H] | 326.1398 | 326.1397 | 311.11542，268.07114，252.04340 | 3.236 | norisocorydine | SZR |
| 21 | 14.9 | C_22_H_22_O_10_ | [M-H] | 445.114 | 445.1151 | 325.07248，297.07736，282.05405 | 4.845 | 4H-Benzopyran-4-one, 6-β-D-glucopyranosyl-5-hydroxy-2-(4-hydroxyphenyl)-7-methoxy- | SZR |
| 22 | 15.03 | C_25_H_24_O_12_ | [M-H] | 515.1195 | 515.1776 | 310.87097，191.05644，174.95633 | 3.286 | 3,5-Dicaffeoyl quinic acid | CX |
| 23 | 15.13 | C_18_H_24_O_10_ | [M-H] | 399.1297 | 399.1307 | 163.04036，145.02974，119.05038 | 5.353 | regaloside A | BH |
| 24 | 15.59 | C_9_H_10_O_2_ | [M-H] | 149.0608 | 149.0611 | 134.03722，120.95514，68.98981 | 9.016 | 2-Methoxy-4-vinylphenol | CX、BH |
| 25 | 15.84 | C_19_H_26_O_11_ | [M-H] | 429.1402 | 429.1484 | 193.05101，175.04036，134.03760 | 2.11 | 4-Hydroxyacetophenone 4-O-(6'-O-beta-D-apiofuranosyl)-beta-D-glucopyranoside | DS |
| 26 | 16.2 | C_16_H_14_O_4_ | [M-H] | 269.0819 | 269.0824 | 254.05904，200.88136，159.04541，109.02969 | 5.926 | Saprionide | DS |
| 27 | 16.23 | C_12_H_14_O_5_ | [M-H] | 237.0768 | 237.0774 | 163.04039，145.02979，119.05042 | 6.875 | 1-o-p-cumaroylglycerol | BH |
| 28 | 16.28 | C_9_H_8_O_3_ | [M-H] | 163.0401 | 163.0403 | 119.05042，69.84799 | 8.399 | trans-p-Coumaric acid | DS、BH |
| 29 | 16.51 | C_27_H_24_O_12_ | [M-H] | 539.1195 | 539.1282 | 297.07718，197.04588，179.03526，161.02469，135.04541 | 2.61 | Yunnaneic acid D | DS |
| 30 | 16.59 | C27H30O15 | [M-H] | 593.1512 | 593.1531 | 413.08884，293.04630，89.02459 | 5.03 | Isovitexin-2″-O-β- D-glucopy-ranoside | SZR |
| 31 | 16.92 | C_14_H_18_O_7_ | [M-H] | 297.098 | 297.1058 | 160.97595，135.04549 | 1.9 | Picein | DS |
| 32 | 17.07 | C_8_H_14_O_2_ | [M-H] | 141.0921 | 141.021 | 114.01013，98.02497 | 19.677 | Hex-3-enyl acetate | BH |
| 33 | 17.12 | C28H32O15 | [M-H] | 607.1668 | 607.1687 | 487.12534，427.10431，324.06482，307.06186，292.03998 | 4.864 | Spinosin | SZR |
| 34 | 17.15 | C_13_H_16_O_6_ | [M-H] | 267.0874 | 267.0881 | 193.05095，175.04030，149.06102，134.03755 | 6.797 | 1-O-feruloylglycerol | BH |
| 35 | 17.25 | C_12_H_16_O_5_ | [M-H] | 239.0925 | 239.093 | 177.09239，170.88338，102.95703，95.05038 | 6.566 | Senkyunolide-R | CX |
| 36 | 17.5 | C_10_H_10_O_4_ | [M-H] | 193.0506 | 193.0511 | 178.02760，149.06116，134.03764，121.02961 | 7.95 | Ferulic Acid | CX、DS |
| 37 | 17.96 | C_24_H_26_O_13_ | [M-H] | 521.1301 | 521.1385 | 323.07806，197.04596，179.03529，161.02472 | 2.22 | Salviaflaside | DS |
| 38 | 18.55 | C_35_H_36_O_17_ | [M-H] | 727.188 | 727.1874 | 727.18738，307.06219，239.05675，179.03543，137.02466 | 2.96 | 6ʹʹʹ-p-Hydroxyl benzoylspinosin | SZR |
| 39 | 18.63 | C_20_H_26_O_11_ | [M-H] | 441.1402 | 441.1409 | 381.12009，163.04042，145.02980 | 3.994 | regaloside B | BH |
| 40 | 19.08 | C_27_H_20_O_12_ | [M-H] | 535.0882 | 535.0895 | 355.04718，311.05685，295.09796，89.02460 | 4.443 | Sagecoumarin | DS |
| 41 | 19.46 | C_37_H_38_O_17_ | [M-H] | 753.2036 | 753.2051 | 607.1687，427.10455，307.06210，205.05106，163.04034，145.02982 | 3.22 | 6ʹʹʹ-p-coumaloylspinosin | SZR |
| 42 | 19.51 | C_28_H_34_O_15_ | [M-H] | 609.1825 | 609.1919 | 301.07269，286.04868 | 3.4 | hesperidin | SZR |
| 43 | 19.57 | C38H40O18 | [M-H] | 783.2142 | 783.2162 | 427.10428，307.06192，235.06174，193.05104 | 4.499 | 6ʹʹʹ-Feruloylspinosin | SZR |
| 44 | 19.87 | C_18_H_16_O_8_ | [M-H] | 359.0772 | 359.085 | 197.04594，179.03528，161.02469，72.99324 | 1.44 | Labiatenic acid | DS |
| 45 | 19.87 | C_36_H_32_O_16_ | [M-H] | 719.1618 | 719.1707 | 197.04590，161.02469，72.99319 | 2.35 | Sagerinic acid | DS |
| 46 | 19.87 | C_17_H_14_O_6_ | [M-H] | 313.0718 | 313.0726 | 161.02472，151.04036，110.97019，88.98824 | 6.086 | Cirsimaritin | DS |
| 47 | 19.9 | C_18_H_14_O_8_ | [M-H] | 357.0616 | 357.0625 | 269.08243，179.03534，161.02475，135.04543，109.02965 | 5.703 | Przewalskinic acid A | DS |
| 48 | 19.9 | C_9_H_6_O_4_ | [M-H] | 177.0193 | 177.0195 | 162.03242，147.96469，133.02969，61.98846 | 6.976 | Aesculetin | DS |
| 49 | 20.05 | C_27_H_22_O_12_ | [M-H] | 537.1038 | 537.1123 | 295.06183，185.02480，109.02970 | 2.1 | Lithospermic acid | DS |
| 50 | 20.56 | C_12_H_18_O_4_ | [M-H] | 225.1132 | 225.1108 | 181.12396，137.09744，125.09739，89.02466 | -5.755 | Senkyunolide-N | CX |
| 51 | 20.84 | C_21_H_20_O_10_ | [M-H] | 431.0984 | 431.1061 | 294.89264，219.03046，201.01988，173.02461，158.97876 | 0.96 | Cosmosiin | DS |
| 52 | 20.84 | C_23_H_32_O_6_ | [M-H] | 403.2126 | 403.2138 | 193.08734，149.06111，135.04535 | 5.543 | Divinatorin D | DS |
| 53 | 20.91 | C_36_H_30_O_16_ | [M-H] | 717.1461 | 717.155 | 339.05154，321.04102，295.06165，185.02461 | 2.31 | Salvianolic acid B | DS |
| 54 | 21.27 | C_20_H_22_O_5_ | [M-H] | 341.1394 | 341.1399 | 326.11679，311.09348，89.02467 | 4.514 | 5-epi-Icetexone | DS |
| 55 | 21.73 | C_18_H_16_O_7_ | [M-H] | 343.0823 | 343.2138 | 197.04587，179.03506，145.02979，135.04541 | 6.512 | Santin | DS |
| 56 | 21.93 | C_15_H_10_O_4_ | [M-H] | 253.0506 | 253.0514 | 235.06232，208.07027，180.07521 | 7.171 | Crysophanol | CX |
| 57 | 22.13 | C_26_H_22_O_10_ | [M-H] | 493.114 | 493.1221 | 295.06174，185.02470，109.02969 | 1.53 | Salvianolic acid A | DS |
| 58 | 22.15 | C_26_H_20_O_10_ | [M-H] | 491.0984 | 491.2291 | 311.05701，293.04639，197.04599，109.02972 | 2.15 | Isosalvianolic acid C | DS |
| 59 | 22.51 | C_19_H_18_O_8_ | [M-H] | 373.0929 | 373.1011 | 174.95639，135.04549 | 2.38 | Methyl rosmarinate | DS |
| 60 | 22.61 | C_28_H_24_O_12_ | [M-H] | 551.1195 | 551.1284 | 321.04129，293.04636，109.02969 | 3 | Methyl melitrate A | DS |
| 61 | 23.1 | C_12_H_16_O_4_ | [M-H] | 223.0976 | 223.0967 | 179.10815，161.09749，137.09738，95.05041 | 0.917 | Senkyunolide I | CX |
| 62 | 24.1 | C_17_H_16_O_3_ | [M-H] | 267.1027 | 267.1035 | 252.07968，145.02977，131.05043 | 7.185 | Danshen spiroketallactone | DS |
| 63 | 24.1 | C_18_H_16_O_5_ | [M-H] | 311.0925 | 311.0573 | 267.10324，174.95648，146.96153 | 7.283 | Tanshindioi C2Przewaquinone E | DS |
| 64 | 24.95 | C_11_H_14_O_2_ | [M-H] | 177.0921 | 177.0924 | 148.96581，132.97252，92.99588，61.98845 | 7.588 | Methyleugenol | CX |
| 65 | 24.95 | C_12_H_14_O_4_ | [M-H] | 221.0819 | 221.0822 | 177.09245，135.04393 | 5.946 | senkyunolide-D | CX |
| 66 | 25.63 | C_10_H_12_O | [M-H] | 147.0815 | 147.0817 | 120.95513，61.98846 | 8.42 | cis-Anethol | BH |
| 67 | 25.88 | C_20_H_20_O_6_ | [M-H] | 355.1187 | 355.1191 | 311.12958，296.10599，281.08249，159.04541 | 4.042 | Cardiophyllidin | DS |
| 68 | 25.96 | C_19_H_18_O_6_ | [M-H] | 341.1031 | 341.1025 | 297.11365，253.12396，216.98964 | 1.599 | Scutellarein 5,6,7,4'-tetramethyl ether | DS |
| 69 | 27.87 | C_11_H_14_O | [M-H] | 161.0972 | 161.0975 | 132.98123，116.92881，83.05049 | 8.929 | Valerophenone | CX |
| 70 | 27.87 | C_12_H_14_O_3_ | [M-H] | 205.087 | 205.0874 | 161.09744，142.92513 | 7.31 | senkyunolide-F | CX |
| 71 | 28.13 | C_20_H_28_O_4_ | [M-H] | 331.1915 | 331.1925 | 287.20236，239.14476，116.92883 | 6.293 | Divinatorin A | DS |
| 72 | 28.18 | C_11_H_16_O | [M-H] | 163.1128 | 163.1132 | 161.0975，93.03478，83.05036 | 8.695 | O-Methylthymol | BH |
| 73 | 28.18 | C_12_H_16_O_3_ | [M-H] | 207.1027 | 207.1031 | 163.11314，121.06630 | 7.384 | Senkyunolide-K | CX |
| 74 | 30.44 | C_12_H_12_O_3_ | [M-H] | 203.0714 | 203.0719 | 174.03241，160.01683， | 7.777 | 3-Butylidene-7-hydroxyphthalide | CX |
| 75 | 31.6 | C_13_H_16_O_3_ | [M-H] | 219.1027 | 219.1032 | 190.98782，175.11313，89.02465 | 7.572 | methyl 2-pentanoylbenzoate | CX |
| 76 | 32.05 | C_16_H_22_O_4_ | [M-H] | 277.1445 | 277.0521 | 256.98306，233.15520，163.11316 | 9.329 | (3Z,6S,7R)-3-butylidene-6-butyryl-7-hydroxy-4,5,6,7-tetrahydroisobenzofuran-1-one | CX |
| 77 | 32.07 | C_20_H_22_O_6_ | [M-H] | 357.1344 | 357.1353 | 327.12463，174.95633，158.97881 | 5.643 | Polystachyne B | DS |
| 78 | 32.28 | C_12_H_16_O_2_ | [M-H] | 191.1078 | 191.1083 | 146.95920，102.94902，61.98849 | 8.34 | Senkyunolide A | CX |
| 79 | 33.64 | C_18_H_16_O_4_ | [M-H] | 295.0976 | 295.0982 | 265.08759，164.92728，158.97882 | 5.844 | Danshenxinkun A | DS |
| 80 | 33.79 | C_12_H_20_O_2_ | [M-H] | 195.1391 | 195.1393 | 130.99272，96.96024，92.92905，61.98847 | 7.09 | Neryl acetate | BH |
| 81 | 34.97 | C_22_H_22_O_6_ | [M-H] | 381.1344 | 380.9951 | 313.14529，269.15527，213.09282，187.07671 | -4.29 | Salvisplendin A | DS |
| 82 | 34.97 | C_19_H_22_O_4_ | [M-H] | 313.1445 | 313.1451 | 269.15533，226.10056，213.12875，187.07675 | 5.443 | Neocryptotanshinone | DS |
| 83 | 36.03 | C_18_H_16_O_3_ | [M-H] | 279.1027 | 279.1033 | 264.07941，251.10831，164.92734 | 6.088 | Danshenxinkun B | DS |
| 84 | 37.59 | C_20_H_26_O_2_ | [M-H] | 297.186 | 297.1865 | 282.16336，160.97575，92.92830 | 5.328 | Przewalskin D | DS |
| 85 | 37.79 | C_18_H_24_O_2_ | [M-H] | 271.1704 | 271.1708 | 259.17044，225.16554，156.99950 | 5.618 | Przewalskin | DS |
| 86 | 39.97 | C_18_H_14_O_3_ | [M-H] | 277.087 | 277.0877 | 249.09283，61.98846 | 6.349 | 1,2-Dihydrotanshinone | DS |
| 87 | 39.99 | C_20_H_28_O_3_ | [M-H] | 315.1966 | 315.1976 | 285.18668，270.94974，234.97287 | 6.849 | Pisiferic acid | DS |
| 88 | 40.53 | C_20_H_28_O_2_ | [M-H] | 299.2017 | 299.2022 | 270.94986，254.95453 | 5.526 | Sugiol | DS |
| 89 | 44.55 | C_21_H_28_O_2_ | [M-H] | 311.2017 | 311.2024 | 174.95639，149.09743，96.96024 | 5.891 | 5,6-Didehydro-O-methylsugiol | DS |
| 90 | 45.5 | C_18_H_34_O_2_ | [M-H] | 281.2486 | 281.2493 | 252.84363，196.02592，166.92445 | 6.269 | oleic acid | CX |
| 91 | 45.91 | C_18_H_32_O_2_ | [M-H] | 279.233 | 279.2336 | 164.92752，148.94890，120.95487 | 6.386 | Linoleic acid | CX |
