# Supplementary material for: Compound identification of Shuangxinfang and its potential mechanisms in the treatment of myocardial infarction with depression: insights from LC-MS/MS and bioinformatic prediction
Source: Front Pharmacol. 2025 Jan 28;16:1499418. doi: 10.3389/fphar.2025.1499418 (PMC11811099; doi:10.3389/fphar.2025.1499418)
Supplement: Supplementary file 2 [file Table2.docx]

Table S2. PCF compounds in the postive ion mode.

| Peaks  no. | tR/min | Molecular formula | Detection mode | Theoretical value | Measured value | Secondary  fragment（MS/MS） | error（ppm） | ingredients | resource |
| --- | --- | --- | --- | --- | --- | --- | --- | --- | --- |
| 1 | 0.76 | C_9_H_10_O_2_ | [M+H] | 151.0754 | 151.0355 | 128.01952，123.04063 | 0.28 | 2-Methoxy-4-vinylphenol | CX、BH |
| 2 | 0.8 | C_12_H_16_O_5_ | [M+H] | 241.1071 | 241.9543 | 112.08738，102.97060 | -3.79 | Senkyunolide-R | CX |
| 3 | 0.96 | C_5_H_5_N_5_ | [M+H] | 136.0618 | 136.0619 | 119.04927，94.06577 | 0.869 | adenine | CX |
| 4 | 0.98 | C_7_H_10_N_2_ | [M+H] | 123.0917 | 123.0405 | 118.03525，100.02487，82.01445 | 1 | trimethylpyrazine | CX |
| 5 | 0.98 | C_7_H_9_NO_2_ | [M+H] | 140.0706 | 140.0344 | 116.97228，112.03976 | 1.146 | 5-(Methoxymethyl)-1H-pyrrole-2-carbaldehyde | DS |
| 6 | 0.99 | C_10_H_13_N_5_O_4_ | [M+H] | 268.104 | 268.1038 | 136.062，97.02899 | -1.009 | adenosine | CX、BH |
| 7 | 1.01 | C_8_H_10_N_2_O | [M+H] | 151.0866 | 151.0754 | 137.02838，119.01814 | 1.35 | 1-Acetyl-2-phenylhydrazine | CX |
| 8 | 1.01 | C_6_H_10_O_5_ | [M+H] | 163.0601 | 163.1049 | 98.06055，85.02905 | -1.29 | Dimethyl D-malate | CX |
| 9 | 1.03 | C_7_H_6_O_2_ | [M+H] | 123.0441 | 123.0556 | 105.03741，96.04488，80.05019 | 11.544 | p-Hydroxybenzaldehyde | BH |
| 10 | 1.03 | C_12_H_22_O_11_ | [M+H] | 343.1235 | 343.1736 | 297.11234，204.01753 | -0.22 | sucrose | CX |
| 11 | 1.03 | C_6_H_6_O_3_ | [M+H] | 127.039 | 127.0392 | 109.02882，99.04460，81.03415，69.03429 | 2.121 | methyl furan-2-carboxylate | CX |
| 12 | 1.08 | C_17_H_26_O_11_ | [M+H] | 407.1548 | 407.1527 | 245.06342，203.05286 | -5.104 | Shanzhiside methyl ester | DS |
| 13 | 1.09 | C_19_H_21_NO_4_ | [M+H] | 328.1543 | 328.1545 | 292.11816，264.12320，166.08643 | 0.443 | norisocorydine | SZR |
| 14 | 1.22 | C_13_H_14_O_3_ | [M+H] | 219.1016 | 219.1741 | 172.95270，154.94194 | 3.39 | Chuanxiongol | CX |
| 15 | 1.42 | C_12_H_14_O_5_ | [M+H] | 239.0914 | 239.0701 | 147.04422，119.04955 | 0.58 | 1-o-p-cumaroylglycerol | BH |
| 16 | 1.42 | C_10_H_14_ | [M+H] | 135.1168 | 135.0667 | 112.0398，93.07067 | 0.87 | Cymol | CX |
| 17 | 1.42 | C_11_H_14_O | [M+H] | 163.1117 | 163.0391 | 145.02856，117.03391 | -0.1 | Valerophenone | CX |
| 18 | 1.42 | C_9_H_8_O_4_ | [M+H] | 181.0495 | 181.0494 | 163.03912，121.06512 | -1.023 | Caffeic acid | DS |
| 19 | 1.42 | C_17_H_12_O_3_ | [M+H] | 265.0859 | 265.104 | 247.13380，175.07574 | 0.48 | Tanshilactone | DS |
| 20 | 1.42 | C_28_H_32_O_15_ | [M+H] | 609.1814 | 609.1748 | 327.08664，198.47313 | 1.1 | Spinosin | SZR |
| 21 | 1.42 | C_16_H_19_NO_8_ | [M+H] | 354.1183 | 374.1552 | 146.06015，127.03915 | -0.02 | N-glc-indoleacectic Acid | SZR |
| 22 | 1.42 | C_17_H_19_NO_3_ | [M+H] | 286.1438 | 286.1438 | 269.11731，237.09114，209.09637，175.07555，107.04960 | 0 | Coclaurine | SZR |
| 23 | 1.42 | C_20_H_23_NO_4_ | [M+H] | 342.17 | 342.1701 | 297.11227，282.08862，265.08603，237.09113，58.06602 | 0.454 | Zizyphusine | SZR |
| 24 | 1.48 | C_15_H_14_O_6_ | [M+H] | 291.0863 | 291.1731 | 139.03915，95.04956 | 1.32 | (-)-Epicatechin | BH |
| 25 | 1.57 | C_12_H_14_O_4_ | [M+H] | 223.0965 | 223.0966 | 205.08533，167.10684 | 0.379 | senkyunolide-D | CX |
| 26 | 1.6 | C_20_H_24_NO_4_ | [M+H] | 343.1778 | 343.1736 | 297.11234，265.08609，58.06603 | -12.238 | Magnoflorine | SZR |
| 27 | 1.61 | C_22_H_22_O_10_ | [M+H] | 447.1286 | 447.13 | 297.07593，207.10220 | 3.191 | 4H-Benzopyran-4-one, 6-beta-D-glucopyranosyl-5-hydroxy-2-(4-hydroxyphenyl)-7-methoxy- | SZR |
| 28 | 1.66 | C_10_H_16_O | [M+H] | 153.1274 | 153.0328 | 130.01691，102.02239 | -4.387 | (-)-Camphor | DS |
| 29 | 1.7 | C_16_H_12_N_2_O_2_ | [M+H] | 265.0972 | 265.0974 | 218.98392，176.07063 | 1.55 | Perlolyrine | CX |
| 30 | 1.7 | C_38_H_40_O_18_ | [M+H] | 785.2287 | 785.4141 | 177.05481，145.02850 | 1.95 | 6ʹʹʹ-feruloylspinosin | SZR |
| 31 | 1.7 | C_37_H_38_O_17_ | [M+H] | 755.2182 | 755.2123 | 147.04416，119.04942 | 1.79 | 6ʹʹʹ-p-coumaloylspinosin | SZR |
| 32 | 1.73 | C_7_H_6_O_3_ | [M+H] | 139.039 | 139.0392 | 111.04449，98.96152 | 1.866 | 4-Hydroxybenzoic acid | DS |
| 33 | 1.77 | C_15_H_15_NO_2_ | [M+H] | 242.1176 | 242.1179 | 147.04419，119.04945 | 0.95 | Prioline | DS |
| 34 | 1.81 | C_20_H_26_O_11_ | [M+H] | 443.1548 | 443.0856 | 147.04416，117.05494 | 0.03 | regaloside B | BH |
| 35 | 1.81 | C_20_H_18_O_10_ | [M+H] | 419.0973 | 419.1871 | 159.04422，139.03908 | 0.6 | Salvianolic acid D | DS |
| 36 | 1.81 | C_9_H_8_O_3_ | [M+H] | 165.0546 | 165.0553 | 147.04424，110.90517 | 4.116 | trans-p-Coumaric acid | DS |
| 37 | 1.81 | C9 H8 O3 | [M+H] | 165.0546 | 165.0553 | 147.04424，119.04949 | 4.116 | 4-Hydroxycinnamic acid | DS |
| 38 | 2.14 | C_8_H_7_NO | [M+H] | 134.06 | 134.0602 | 106.06561，92.95015，79.05490 | 1.489 | 2-Methylbenzoxazol | CX |
| 39 | 2.33 | C_10_H_10_O_4_ | [M+H] | 195.0652 | 195.0624 | 177.05486，135.04434 | -14.177 | Ferulic acid | DS、BH |
| 40 | 2.47 | C_36_H_30_O_16_ | [M+H] | 719.1607 | 719.1536 | 383.09320，139.03906 | 0.3 | Lithospermic acid B | DS |
| 41 | 13.22 | C_12_H_16_O_3_ | [M+H] | 209.1172 | 209.1175 | 153.05481，145.10126 | 1.143 | Senkyunolide-K | CX |
| 42 | 13.46 | C_12_H_18_O_4_ | [M+H] | 227.1278 | 227.1758 | 153.05476，135.11696 | 0.2 | Senkyunolide-N | CX |
| 43 | 18.18 | C_12_H_16_O_4_ | [M+H] | 225.1121 | 225.1955 | 207.10173，165.09106 | 0.69 | Senkyunolide I | CX |
| 44 | 18.2 | C_12_H_14_O_3_ | [M+H] | 207.1016 | 207.1019 | 189.09117，179.10667 | 1.348 | senkyunolide-F | CX |
| 45 | 19.49 | C_11_H_14_O_2_ | [M+H] | 179.1067 | 179.0641 | 137.08231，119.08583 | 0.87 | Methyleugenol | CX |
| 46 | 19.64 | C_10_H_8_O_3_ | [M+H] | 177.0546 | 177.054 | 159.04424，135.94545 | -3.393 | Herniarin | DS |
| 47 | 19.71 | C_26_H_20_O_10_ | [M+H] | 493.1129 | 493.1063 | 267.06531，139.03908 | 1.41 | Isosalvianolic acid C | DS |
| 48 | 19.74 | C_15_H_10_O_4_ | [M+H] | 255.0652 | 255.0653 | 204.93327，158.92786 | 0.293 | Crysophanol | CX |
| 49 | 19.88 | C_35_H_58_O_6_ | [M+H] | 575.4306 | 575.426 | 114.09174，96.08127 | 4.55 | Stigmasta-5,22-dien-3-O-.beta.-D-glucopyranoside | BH |
| 50 | 21.89 | C_12_H_12_O_3_ | [M+H] | 205.0859 | 205.0865 | 149.02348，113.96442 | 2.824 | 3-Butylidene-7-hydroxyphthalide | CX |
| 51 | 22.98 | C_18_H_16_O_5_ | [M+H] | 313.1071 | 313.1454 | 267.10159，221.09621 | 0.09 | Tanshindioi C2Przewaquinone E | DS |
| 52 | 23.95 | C_12_H_12_O_2_ | [M+H] | 189.091 | 189.091 | 161.09621，119.08583 | -0.191 | 3-Butylidenephthalide | CX |
| 53 | 27.23 | C_19_H_20_O_4_ | [M+H] | 313.1434 | 313.1432 | 295.13290，266.93121 | -0.784 | Miltionone I | DS |
| 54 | 28.14 | C_18_H_12_O_4_ | [M+H] | 293.0808 | 293.0807 | 249.09109,193.10129 | -0.598 | Tanshinol A | DS |
| 55 | 29.36 | C_18_H_14_O_4_ | [M+H] | 295.0965 | 295.0925 | 249.09116，100.02487 | -13.506 | 3-Hydroxymethylenetanshinquinone | DS |
| 56 | 31.87 | C_20_H_22_O_6_ | [M+H] | 359.1489 | 359.2184 | 263.10678，217.10107 | -0.23 | Polystachyne B | DS |
| 57 | 32.04 | C_19_H_18_O_4_ | [M+H] | 311.1278 | 311.1269 | 275.10666，251.10670，181.10080 | -2.878 | Przewaquinone A | DS |
| 58 | 32.57 | C_8_H_8_O_2_ | [M+H] | 137.0597 | 137.0598 | 91.05483，77.03928 | 0.685 | Clorius | BH |
| 59 | 32.57 | C_12_H_16_O_2_ | [M+H] | 193.1223 | 193.1224 | 137.05983，119.08588 | 0.433 | Senkyunolide A | CX |
| 60 | 33.39 | C_18_H_16_O_4_ | [M+H] | 297.1121 | 297.1124 | 261.09113，233.09636，169.06520 | 0.789 | Danshenxinkun A | DS |
| 61 | 33.44 | C_19_H_16_O_4_ | [M+H] | 309.1121 | 309.1118 | 265.12250，207.09995 | -1.118 | Tanshinaldehyde | DS |
| 62 | 33.59 | C_20_H_20_O_5_ | [M+H] | 341.1384 | 341.1382 | 235.11185，163.11163 | -0.411 | Salvifaricin | DS |
| 63 | 34.18 | C_19_H_24_O_2_ | [M+H] | 285.1849 | 285.1486 | 256.92868，226.89156 | 0.117 | Salvirecognone | DS |
| 64 | 35.56 | C_11_H_16_ | [M+H] | 149.1325 | 149.0235 | 105.96339，88.07622 | 1.44 | Amylbenzene | CX |
| 65 | 35.56 | C_12_H_16_O | [M+H] | 177.1274 | 177.1274 | 149.13266，120.08114 | 0.047 | Hexaphenone | CX |
| 66 | 35.59 | C_12_H_18_O_2_ | [M+H] | 195.138 | 195.138 | 149.13271，143.07066，79.05494 | 0.326 | Cnidilide | CX |
| 67 | 35.73 | C_12_H_14_O_2_ | [M+H] | 191.1067 | 191.1068 | 173.09628，163.11163，135.04425，117.07022 | 0.491 | (Z)-Ligustilide | CX |
| 68 | 35.77 | C_18_H_14_O_3_ | [M+H] | 279.1016 | 279.1017 | 261.09122，205.10135 | 0.534 | 1,2-Dihydrotanshinone | DS |
| 69 | 36.57 | C_18_H_24_O_2_ | [M+H] | 273.1849 | 273.1848 | 199.11200，171.08057 | -0.573 | Przewalskin | DS |
| 70 | 36.84 | C_18_H_16_O_3_ | [M+H] | 281.1172 | 281.1173 | 235.11192，164.92055 | 0.424 | Danshenxinkun B | DS |
| 71 | 37.23 | C_19_H_22_O_4_ | [M+H] | 315.1591 | 315.159 | 297.14865，237.09039 | -0.367 | Neocryptotanshinone | DS |
| 72 | 37.4 | C_20_H_26_O_2_ | [M+H] | 299.2006 | 299.2007 | 229.12253，187.07538， | 0.312 | Przewalskin D | DS |
| 73 | 37.74 | C_20_H_18_O_5_ | [M+H] | 339.1227 | 339.1156 | 261.09113，233.09622，189.07082 | 0.38 | Methyl tanshinonate | DS |
| 74 | 38.37 | C_17_H_16_O_3_ | [M+H] | 269.1172 | 269.117 | 251.10675，223.11179 | -0.709 | Danshen spiroketallactone | DS |
| 75 | 39.79 | C_18_H_12_O_3_ | [M+H] | 277.0859 | 277.0861 | 262.06284，207.08023 | 0.611 | Tanshinone I | DS |
| 76 | 39.83 | C_20_H_28_O_3_ | [M+H] | 317.2111 | 317.2092 | 215.10684，173.05965 | -6.057 | Pisiferic acid | DS |
| 77 | 39.86 | C_19_H_20_O_3_ | [M+H] | 297.1485 | 297.1487 | 279.13794，251.14316，213.10271 | 0.434 | Cryptotanshinone | DS |
| 78 | 40.23 | C_18_H_30_O_2_ | [M+H] | 279.2319 | 279.0935 | 80.94852，67.05502 | 1.04 | 7alpha-Hydroxy-14,15-dinor-8(17)-labden-13-one | DS |
| 79 | 40.35 | C_20_H_28_O_2_ | [M+H] | 301.2162 | 301.2164 | 283.20673，213.12749 | 0.542 | Sugiol | DS |
| 80 | 41.03 | C_18_H_16_O_2_ | [M+H] | 265.1223 | 265.1224 | 223.07555，167.08609 | 0.391 | 12-Deoxydanshenxinkun B | DS |
| 81 | 42.18 | C_19_H_16_O_3_ | [M+H] | 293.1172 | 293.1172 | 275.10666，247.11176，167.08597 | -0.003 | △1-dehydrotanshinone | DS |
| 82 | 42.97 | C_24_H_30_O_4_ | [M+H] | 383.2217 | 383.2216 | 191.10683，175.11183 | -0.25 | Senkyunolide-P | CX |
| 83 | 42.97 | C_20_H_22_O_3_ | [M+H] | 311.1642 | 311.164 | 283.1694，237.09125 | -0.55 | 6-Methylcryptotanshinone | DS |
| 84 | 43.1 | C_16_H_22_O_4_ | [M+H] | 279.1591 | 279.0928 | 149.02357，135.64874 | 0.12 | (3Z,6S,7R)-3-butylidene-6-butyryl-7-hydroxy-4,5,6,7-tetrahydroisobenzofuran-1-one | CX |
| 85 | 43.52 | C_19_H_20_O_2_ | [M+H] | 281.1536 | 281.1537 | 253.15875，221.09631 | 0.297 | Dehydromiltirone | DS |
| 86 | 43.73 | C_25_H_32_O_5_ | [M+H] | 413.2323 | 413.2253 | 191.10684，135.04413 | 0.88 | wallichilide | CX |
| 87 | 44.03 | C_19_H_18_O_3_ | [M+H] | 295.1329 | 295.133 | 277.12244，207.08104 | 0.437 | Tanshinone IIA | DS |
| 88 | 44.25 | C_24_H_28_O_4_ | [M+H] | 381.206 | 381.206 | 191.10683，135.04417 | -0.12 | Levistolid A | CX |
| 89 | 44.64 | C_18_H_20_O_2_ | [M+H] | 269.1536 | 269.1538 | 254.13028，199.07538 | 0.645 | Carnosol | DS |
| 90 | 45.01 | C_19_H_22_O_3_ | [M+H] | 299.1642 | 299.1642 | 253.15877，183.08057，109.10123 | 0.03 | Neocryptotanshinone II | DS |
| 91 | 45.33 | C_19_H_22_O_2_ | [M+H] | 283.1693 | 283.1694 | 265.15881，223.11186，164.92101 | 0.401 | Miltirone | DS |
| 92 | 46.78 | C_8_H_12_N_2_ | [M+H] | 137.1073 | 137.1073 | 121.07597，96.05620 | 0.037 | tetramethylpyrazine | CX |

DS: *Salvia miltiorrhiza* Bunge [Lamiaceae; *Salviae miltiorrhizae* radix et rhizoma]; CX: the roots and rhizomes of *Chuanxiong Rhizoma* [Umbelliferae; *Ligusticum chuanxiong* hort]; BH: the bulb of *Lilium pumilum* DC [Liliaceae; Lilii Bulbus]; SZR: dried seeds of *Ziziphi Spinosae Semen* [Rhamnaceae; *Ziziphus jujuba* Mill.var.spinosa (Bunge) Hu ex H.F.Chou].
